# Thai Guideline for Nuclear Medicine Investigations of Neurocognitive Disorders: Nuclear Medicine Society of Thailand, the Neurological Society of Thailand, and Thai Medical Physicist Society Collaboration

**DOI:** 10.3390/diagnostics14222474

**Published:** 2024-11-06

**Authors:** Tawika Kaewchur, Tanyaluck Thientunyakit, Wichana Chamroonrat, Benjapa Khiewvan, Peerapon Kiatkittikul, Nantaporn Wongsurawat, Chanisa Chotipanich, Yuttachai Likitjaroen, Vorapun Senanarong, Panya Pasawang, Tanawat Sontrapornpol, Nucharee Poon-iad, Sasithorn Amnuaywattakorn, Supatporn Tepmongkol

**Affiliations:** 1 Division of Nuclear Medicine, Department of Radiology, Faculty of Medicine, Chiang Mai University, Chiang Mai 50200, Thailand; tawika.k@cmu.ac.th (T.K.); 2 PET/CT and Cyclotron Center, Center for Medicine Excellence, Faculty of Medicine, Chiang Mai University, Chiang Mai 50200, Thailand; 3 Division of Nuclear Medicine, Department of Radiology, Faculty of Medicine Siriraj Hospital, Mahidol University, Bangkok 10700, Thailand; stanyalu@hotmail.com (T.T.); benjapa.khi@mahidol.ac.th (B.K.); fortune_nucharee_si@hotmail.com (N.P.-i.); 4 Division of Nuclear Medicine, Department of Diagnostic and Therapeutic Radiology, Faculty of Medicine Ramathibodi Hospital, Mahidol University, Bangkok 10400, Thailand; wichana.cha@mahidol.edu (W.C.); amsasithorn@gmail.com (S.A.); 5 National Cyclotron and PET Centre, Chulabhorn Hospital, Chulabhorn Royal Academy, Bangkok 10210, Thailand; peerapon.kia@cra.ac.th (P.K.); chanisa.ja@gmail.com (C.C.); 6 Division of Nuclear Medicine, Department of Radiology, Faculty of Medicine, Khon Kaen University, Khon Kaen 40002, Thailand; nantwo@kku.ac.th; 7 Department of Medicine, Faculty of Medicine, Chulalongkorn University and King Chulalongkorn Memorial Hospital, Thai Red Cross Society, Bangkok 10330, Thailand; yuttachai.l@chula.ac.th; 8 Department of Internal Medicine, Faculty of Medicine, Siriraj Hospital, Mahidol University, Bangkok 10700, Thailand; vorasenanarong@yahoo.com; 9 Division of Nuclear Medicine, Department of Radiology, King Chulalongkorn Memorial Hospital, Bangkok 10330, Thailand; ppasawang@gmail.com (P.P.); santi_s@hotmail.com (T.S.); 10 Division of Nuclear Medicine, Department of Radiology, Faculty of Medicine, Chulalongkorn University, Bangkok 10330, Thailand; 11 Chulalongkorn University Biomedical Imaging Group (CUBIG), Faculty of Medicine, Chulalongkorn University, Bangkok 10330, Thailand; 12 Chula Neuroscience Center, King Chulalongkorn Memorial Hospital, Thai Red Cross Society, Bangkok 10330, Thailand; 13 Center of Excellence in Cognitive Impairment and Dementia, Faculty of Medicine, Chulalongkorn University, Bangkok 10330, Thailand

**Keywords:** positron emission tomography (PET), single-photon emission computed tomography (SPECT), dementia, neurocognitive disorders, guidance

## Abstract

Nuclear medicine investigations play a significant role in diagnosing dementia, mainly using imaging techniques such as positron emission tomography (PET) and single-photon emission computed tomography (SPECT). By providing functional and molecular data via brain imaging, nuclear medicine investigations offer valuable insights that complement clinical evaluations and structural imaging in the early detection, diagnosis, and differentiation of various types of dementia, leading to more accurate diagnosis and personalized treatment planning. Therefore, the Nuclear Medicine Society of Thailand, the Neurological Society of Thailand, and the Thai Medical Physicist Society have collaborated to establish these practical nuclear medicine investigation guidelines aiming to (1) identify the role of nuclear medicine studies in patients with neurocognitive disorders; (2) assist referrers in requesting the most appropriate procedure for diagnosis of each type of neurocognitive disorders; and (3) identify scientific evidence that is useful to assisting nuclear medicine professionals in recommending, performing, interpreting, and reporting the results of nuclear medicine investigations in patients with neurocognitive disorders.

## 1. Introduction

Dementia is a syndrome of cognitive decline in different domains. Regarding the etiologies, neurodegenerative brain disease is the most common cause of dementia in the elderly, followed by cerebrovascular diseases. The clinical diagnosis of neurodegenerative brain disease causing dementia is based on clinical criteria [1]. Various neurodegenerative diseases causing dementia have been defined by their clinical syndromes and neuropathological findings, such as Alzheimer’s disease, dementia with Lewy bodies, frontotemporal lobar degeneration, and their variants. The definite diagnosis of neurodegenerative brain disease needs the findings of pathological protein accumulation and neuronal degeneration within the brain parenchyma. Each disease has a specific pattern of brain atrophy and type of accumulated protein, which is revealed during an autopsy [2,3,4]. Nonetheless, obtaining brain tissue for diagnosis is impractical due to its invasiveness.

Previously, brain imaging in dementia, including computed tomography (CT) and magnetic resonance imaging (MRI), was used to rule out reversible causes of dementia, such as cerebrovascular disease, hydrocephalus, tumors, etc. Although modern high-resolution MRI can precisely demarcate gray matter, white matter, and other non-brain parenchymal structures, leading to the detection of gray and white matter change patterns in individuals with neurodegenerative brain disease, molecular imaging such as single-photon emission computed tomography (SPECT) and positron emission tomography (PET) further allow indirect observation of the pattern of functional brain decline or pathological protein accumulation, which are more specific to the disease. The overlaps among clinical syndromes, pathological protein accumulation, and genetic risk are the challenges of dementia diagnosis. Thus, various imaging techniques have been added to diagnostic criteria to improve dementia care in clinical and dementia research [5,6,7,8].

Since the number of dementia patients in Thailand is increasing along with the aging society, there are rising demands for dementia investigations. (In Thailand, accessibility to MRI, SPECT, and PET is still limited, and the cost of these imaging studies is high. Therefore, it is necessary to have guidelines for choosing the most appropriate diagnostic techniques for an individual patient). These guidelines aim to assist clinicians and nuclear medicine specialists in diagnosing, choosing appropriate nuclear medicine investigations, performing image acquisition, interpreting images, and being aware of potential pitfalls in using SPECT and PET in neurocognitive disorders.

## 2. Definitions

Dementia is a syndrome of insidious cognitive decline in one or more domains that is significant from the previous level of cognition. The decline must be severe enough to interfere with occupational, social, or daily life functions. According to the fifth version of the Diagnostic and Statistical Manual of Mental Disorders version 5 (DSM5), revised by the American Psychiatric Association (APA) in 2013 [9], the term major neurocognitive disorder is used as an alternative name for dementia. Mild cognitive impairment (MCI) is also a syndrome of cognitive decline with less severity that does not interfere with occupational, social, or daily life functions. MCI was introduced in order to define the pre-symptomatic dementia [10]. Mild neurocognitive disorder is an alternative term for MCI according to DSM5.

Moreover, MCI has been declared as a disease. In addition, the cognitive domains have been revised and renamed as complex attention, executive function, learning and memory, language, perceptual-motor, and social cognition [9,11]. The causes of dementia can result from various diseases. Each type of dementia has a specific clinical syndrome, diagnostic criteria, brain pathologic findings, and structural and functional change patterns. This information must be assessed before choosing the appropriate investigations. These guidelines discuss the main roles of molecular imaging (SPECT and PET) in assisting in clinical diagnosis and management of neurocognitive disorders associated with neurodegenerative diseases (Table 1).

## 3. Objectives

1. To identify the role of nuclear medicine studies in patients with neurocognitive disorders.
2. To assist referrers in requesting the most appropriate procedure for the diagnosis of each type of neurocognitive disorder.
3. To identify scientific evidence that is useful in assisting nuclear medicine professionals in recommending, performing, interpreting, and reporting the results of nuclear medicine investigations in patients with neurocognitive disorders.

## 4. Guideline Development and Recommended Flow of Nuclear Medicine Investigations of Dementia Syndrome

**Scheme 1:**
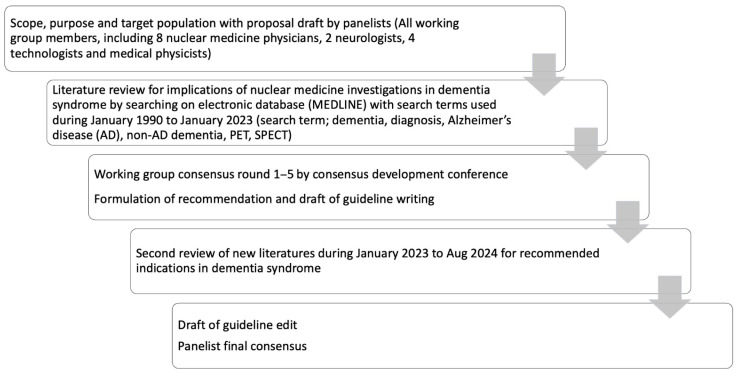
Guideline development process.

The panelists for this guideline development are nuclear medicine specialists who are representatives from Nuclear Medicine Society of Thailand (S.T., T.K., B.Kh., W.C., T.T., N.W., Ch.C., and P.K.), medical physicists/technologists from Thai Medical Physicist Society (P.P., T.S., N.P, and S.A.), and neurologists from The Neurological Society of Thailand and Dementia Association of Thailand (Y.L. and V.S.). The guideline development process is shown in Scheme 1.

**Scheme 2:**
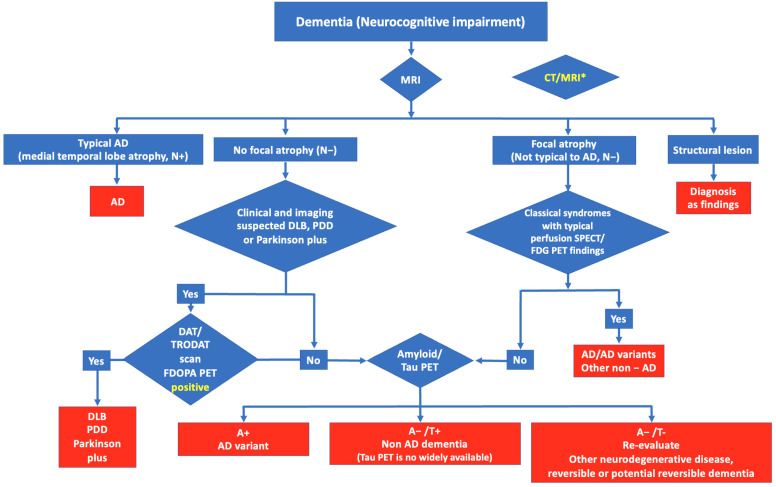
The flow of nuclear medicine investigations in dementia syndrome. MRI = Magnetic resonance imaging; CT = Computed tomography; SPECT = Single-photon emission computed tomography; FDG PET = ^18^F-Fluorodeoxyglucose positron emission tomography; FDOPA PET = ^18^F-Fluoro-L-dopa positron emission tomography; AD = Alzheimer’s disease; DLB = Dementia with Lewy bodies; PDD = Parkinson’s disease dementia; A = Amyloid; T = Tau; N = Neurodegeneration. Note: * = CT is optional if MRI is not available.

The flow of imaging investigation in dementia syndrome is shown in Scheme 2, and the summarized implications of nuclear medicine investigations in dementia syndrome with the level of evidence are listed below in Table 2 [12,13,14,15,16,17,18,19,20,21,22,23,24,25,26,27,28,29,30,31,32,33,34].

Relative contraindications for nuclear medicine investigations are pregnancy/breastfeeding and the patients who are unable to cooperate.

## 5. Nuclear Medicine Imaging Procedures

### 5.1. Patient Preparation

The patient preparation for nuclear medicine investigations in dementia syndrome is shown in Table 3 [36,37,38,39,40].

### 5.2. Recommended Radiopharmaceutical Dosage

The recommended radiopharmaceutical dosage is shown in Table 4 [36,37,38,39,40,41,42,43,44].

### 5.3. Radiation Dosimetry

Radiation dosimetry in SPECT [36,37] and PET studies [39,41,42,43,44,45,46] is shown in Table 5 and Table 6, respectively.

### 5.4. Brain Perfusion SPECT Acquisition and Image Reconstruction Parameter

The brain perfusion SPECT and SPECT/CT acquisition and reconstruction parameters are shown in Table 7 [37,47].

### 5.5. PET Acquisition and Image Reconstruction Parameter

The brain PET acquisition and reconstruction parameters are shown in Table 8 [38,39,40,48,49].

## 6. Imaging Data Display, Image Interpretation, and Reporting Format

### 6.1. Brain Perfusion SPECT and FDG PET

#### 6.1.1. Imaging Data Display [45,50]

The display plane for SPECT and PET is the AC–PC plane (parallel to the line passing from the anterior commissure to the posterior commissure, which is approximately the line passing through the anterior and posterior poles of the brain in a sagittal image).

Resliced axial images are usually used for interpretation, while coronal and sagittal images help to better delineate abnormal regions.

A continuous gray or color display scheme could be selected according to the reader’s preference.

The surface projection technique with comparison to a normal age-matched database by Z-score using three-dimensional stereotactic surface projections (3D-SSP) or Neurostat further avoids misdiagnosis of dementia type made by visual analysis alone. It improves diagnostic confidence for brain perfusion SPECT and FDG-PET, especially in non-experienced readers [51,52]. Other programs, such as eZIS [53] that compare the patient’s data with or without a normal database can also be used depending on the user’s preferences.

Three-dimensional stereotactic surface projection (3D-SSP) is software for neurological image analysis, which compares a patient’s brain to an age-matched normal database voxel-by-voxel with normalization of the count to the brain region that is usually not affected by the disease process, e.g., thalamus, cerebellum, and pons. Global brain (whole gray matter) for reference is optional.

The easy Z-score imaging system, or eZIS [53], combines statistical parametric mapping (SPM) and 3D-SSP. Instead of using a normal database for robust statistical imaging analysis, eZIS can utilize SPM processing in normalization, smoothing, and image conversion functions for statistical analysis. Furthermore, if a normal database is available, even from different institutes or different cameras/SPECTs, it can also be used.

**Figure 1:**
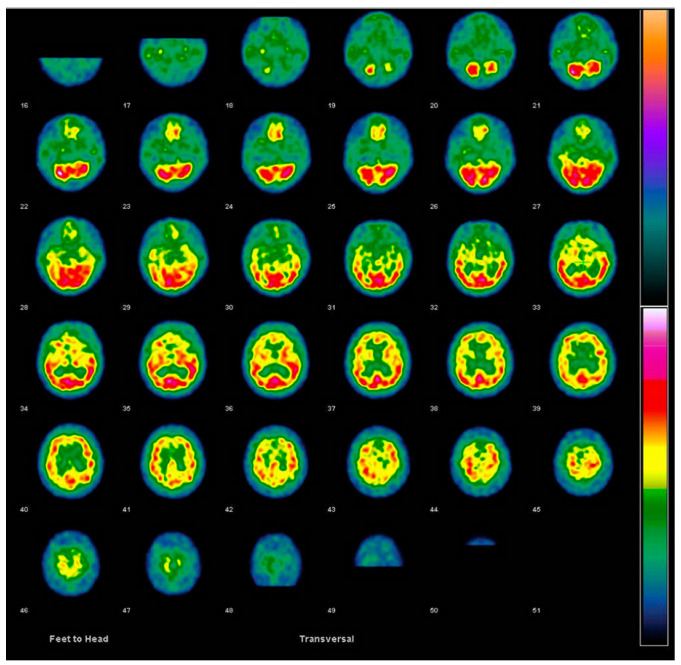
Brain ECD SPECT in frontotemporal dementia displayed in rainbow color scale in the axial AC–PC plane.

**Figure 2:**
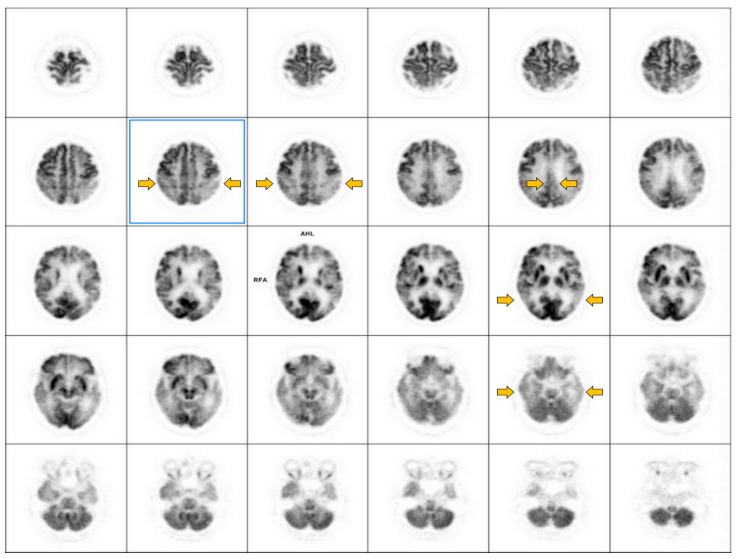
Brain FDG-PET in Alzheimer’s disease displayed in the axial AC–PC plane. Hypometabolism at posterior cingulate gyri, precunei, bilateral parietal and temporal cortices (yellow arrows), with relatively preserved metabolism at bilateral primary sensorimotor areas, visual cortices, basal ganglia, thalami, and cerebelli are observed. (Black shows higher metabolism and gray to white show lower metabolism).

Examples of brain perfusion SPECT and brain FDG-PET image display for visual analysis in Alzheimer’s disease are demonstrated in Figure 1 and Figure 2, respectively.

#### 6.1.2. Image Interpretation for Brain Perfusion SPECT and FDG-PET

Since brain perfusion tracers, e.g., ^99m^Tc ECD and brain glucose metabolism PET radiopharmaceutical (^18^F-FDG), are taken up by neuronal cells, depending on synaptic activity [54,55,56], their uptake thus also represents areas of viable cells. Dementia, which has synaptic dysfunction and neuronal death, can be imaged with these two types of imaging. Areas of hypoperfusion/hypometabolism are the areas to look for in dementia. Each type of dementia shows specific patterns of hypoperfusion/hypometabolism in the cortex, as shown in Table 9 [57,58,59,60,61,62,63].

**Figure 3:**
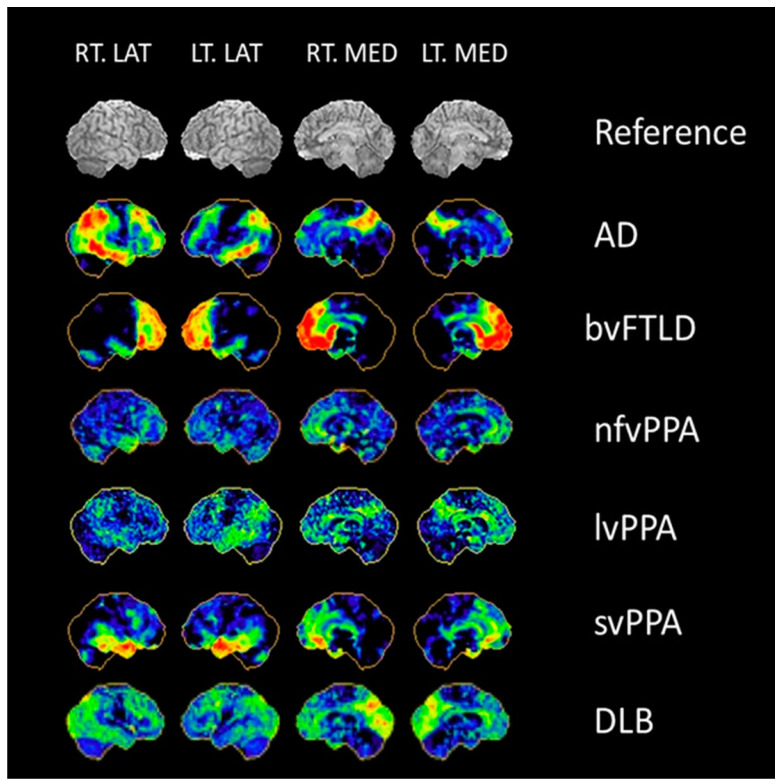
3D-SSP of dementia syndrome. Courtesy: Professor Satoshi Minoshima, modified with permission. LT = left, RT = right, LAT = lateral view, MED = medial view, AD = Alzheimer’s disease, bvFTLD = behavioral variant FTD, nfvPPA = non-fluent variant primary progressive aphasia, lvPPA = logopenic variant primary progressive aphasia, svPPA = semantic variant primary progressive aphasia, DLB = dementia with Lewy bodies. Color shows the specific hypometabolic brain region in each type of dementia.

Using 3D-SSP, the hypometabolic regions affected in each type of dementia are more evident, as shown in Figure 3.

#### 6.1.3. Reporting Format

Each report must include specific details, such as the patient’s identification, the referring clinician, the date and time of the study, and the reporting physician’s signature for quality assurance purposes. The reporting physician must ensure these details are accurately matched with the main content of the report. The suggested structure for reports on brain SPECT and brain PET [36,37,45,64,65] consists of four key sections.

- History: The relevant history should be noted along with an indication of the study, for example, clinical cognitive impairment and other related disorders, type of suspected dementia, duration of symptom, recent medication, and findings of other related studies (e.g., neuropsychological test, CT, and MRI).
- Techniques:
  1. Radiopharmaceutical type and dosage.
  2. Uptake time: after tracer injection to image acquisition.
  3. Describe imaging and processing techniques, including imaging quality and limitations. These should also be thoroughly detailed if specific software or anatomical co-registration methods are used.
  4. Ancillary drugs (if used), e.g., type and time of sedative drugs.
  5. Fasting duration and serum glucose level for FDG PET.
  6. Type of additional software, e.g., 3D-SSP and normal age range and ethnicity, used for analysis.
- Findings:
  - SPECT or PET hypoperfusion/hypometabolic regions should be mentioned. Its location, extension, and severity should be reported.
  - Semiquantitative results (if done) e.g., Z-score comparison, can be described.
  - Correlative imaging findings, if available, e.g., MRI and CT, should be mentioned
- Interpretations/Impressions/Conclusions: Conclude whether the hypoperfusion/hypometabolic pattern found matches with the type of dementia. The final impression from imaging data should be interpreted along with available clinical and correlative imaging data. If they seem discordant, direct discussion with the multidisciplinary team would be the best (if possible) or provide further recommendations.

### 6.2. Brain Amyloid PET

#### 6.2.1. Imaging Data Display [39]

The pixel size of at least 16-bit pixels.

Imaging plane: The alignment for displaying the tomographic images is the AC–PC line. The transaxial images are mainly used for image interpretation. However, coronal and sagittal images are also helpful in differentiating the distribution of the radiotracer in gray matter from subcortical white matter and confirming that the entire brain has been reviewed.

The color scale should be specific for the radiopharmaceutical used, as follows:

Gray scale: ^18^F-florbetaben
Color (Rainbow): ^11^C-PiB, ^18^F-flutemetamol and ^18^F-NAV4694
Reverse gray scale: ^18^F-florbetapir
Maximum intensity of the display scale
^18^F-florbetapir: the brightest region of overall brain uptake
^18^F-florbetaben: the white matter maximum
^18^F-flutemetamol: setting the scale intensity in the pons region to 90%

#### 6.2.2. Image Interpretation for Brain Amyloid PET

##### Visual Analysis [39,66,67,68]

There are different criteria for interpreting amyloid PET images among radiotracers, and interpreters should know the recommendations specific to a given amyloid tracer (Table 10). However, there are fundamental principles that can be applied. A systematic review of transaxial amyloid PET images starts at the cerebellar level. Usually, the cerebellar cortices are free from beta-amyloid deposition. Therefore, this level references normal cortical activity and gray–white matter contrast. After that, it is recommended to scroll up to review the entire cerebral cortical and subcortical regions with particular attention to the frontal, lateral temporal, posterior cingulate/precuneus, parietal cortices, and the basal ganglia. The visual analysis comprises a binary interpretation as a positive or negative scan.

##### Negative Scan

**Figure 4:**
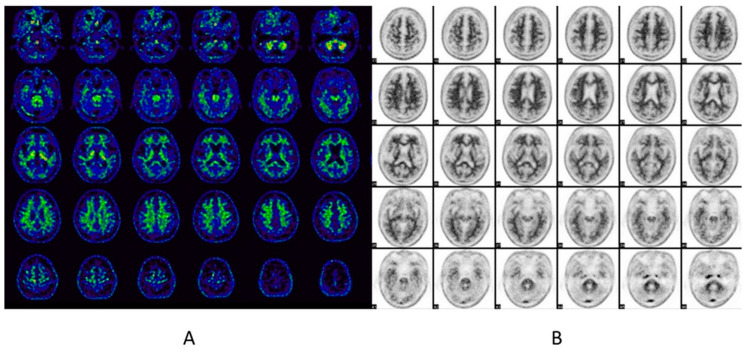
Example images of negative scans for beta-amyloid deposition acquired from (**A**) ^11^C-PiB and (**B**) ^18^F-florbetapir.

Normal distribution for beta-amyloid radiopharmaceuticals is predominantly in the white matter with no or little retention in the gray matter. With the clear distinction of radiotracer uptake between gray and white matter, there is a nicely seen contrast between grey and white matter. In ^11^C-PiB, some radiotracer uptake within the cerebral cortices can be expected but not exceed that in the adjacent white matter. The mechanism of white matter uptake is considered non-specific and varies among radiotracers. The negative uptake pattern resembles a blueprint of white matter distribution, i.e., a white matter sulcal pattern, with numerous concave arboreal ramifications. There should be a clear, wide, irregular gap between the cerebral hemispheres. Example images of negative scans are shown in Figure 4.

##### Positive Scan

**Figure 5:**
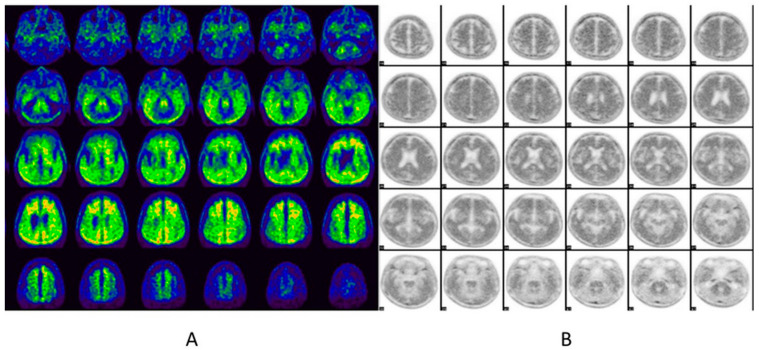
Example images of positive scans for beta-amyloid deposition acquired from (**A**) ^11^C-PiB and (**B**) ^18^F-florbetapir.

A positive scan is defined as increased retention of tracer radiopharmaceutical uptake in the cerebral cortices, with the same or higher intensity as compared to the uptake in the white matter, and it forms a smooth, regular boundary. The typical brain regions involved are the frontal cortex, medial and lateral posterior parietal cortices, precuneus, occipital cortex, lateral temporal cortices, and striatum (most notably at the caudate head). By contrast, the sensorimotor and visual cortex can be relatively preserved. A typical finding in a positive ^11^C-PiB scan is the intense retention in the cerebral cortices (cortical ribbon), which is higher than in the white matter. This finding is less often seen in ^18^F-labeled radiopharmaceuticals, which is generally considered a positive scan when loss of the normal white matter pattern or loss of the gray matter and white matter contrast. Example images of negative scans are shown in Figure 5. However, there are differences in details regarding the criteria used to define amyloid positivity among radiopharmaceuticals. The brief criteria for brain amyloid positivity for each radiopharmaceutical are summarized in Table 10.

Please note that positive amyloid PET can be found in other conditions apart from Alzheimer’s disease, including dementia with Lewy bodies (DLB), Parkinson’s disease dementia (PDD), and cerebral amyloid angiopathy (CAA). In the elderly population with normal cognition, the incidence of amyloid positivity also increases with age [69,70].

In some complex cases, the available CT or fused PET/CT images or coregistration of PET and MRI images can provide helpful information on localization (gray vs. white matter) of radiotracer uptake. Structural MRI also potentially contributes to the differential diagnosis of dementia type, both in amyloid PET positive and negative cases [71,72].

##### Quantification

Quantification analysis to obtain numeric data of amyloid PET images can support visual analysis objectively, assess temporal change, and compare individual PET results with those of the normal control population. Absolute quantitative measurements of amyloid tracer deposition using a dynamic PET imaging protocol and tracer kinetic analysis are not required clinically but may be used for research. However, some semiquantitative analytical methods, namely the SUVR, Centiloid scale, and 3D-SSP, can be performed using available computer-aided analysis software for the clinic.

- Standardized uptake value ratio (SUVR)

The SUVR is a semiquantitative analysis determined by the ratio between the SUV of the cortical region of interest divided by the SUV of the specific reference region recommended for each radiopharmaceutical. Several cortical brain regions are used to assess the SUVR: the global cerebral cortex, frontal, temporal, parietal, precuneus, anterior/posterior cingulate, and composite region, which combines several regional cortices. The brain regions suggested as reference regions are known to be spared from beta-amyloid deposition, either cerebellum (whole or cortex) or pons. However, the cut-off value of the SUVR for amyloid positivity has yet to be agreed upon [68,73,74].

b. Centiloid scale (CL) [75,76,77,78]

The Centiloid scale was proposed to overcome the limitation of using different radiopharmaceuticals to assess beta-amyloid deposition across centers, which makes it difficult for comparison purposes and in multicenter research trials. The concept of this quantification method is to translate the SUVR in the same regions of the brain obtained from each radiopharmaceutical into the common number, called the Centiloid, which ranges from 0 to 100, using the standard formula created from the previous research to assess the association between regional SUV data from each radiopharmaceutical and reference radiopharmaceutical (^11^C-PiB). The average SUVR from cortical VOIs to be used for calculating the CL value are the frontal combination (consisting of dorsolateral prefrontal, ventrolateral prefrontal, and orbitofrontal regions), superior parietal, lateral temporal, lateral occipital, anterior/posterior cingulate, and precuneus.

**Figure 6:**
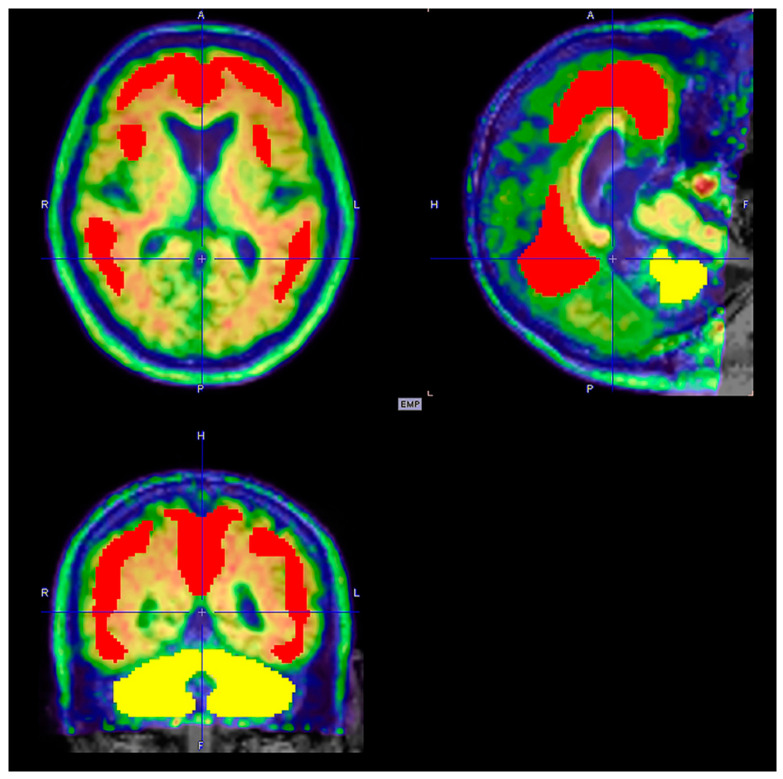
Example of cortical VOIs (red) of beta-amyloid PET images co-registered with the MRI template for assessing the composite SUVR with the whole cerebellum (yellow) as the reference region. The SUVR will be used to calculate the Centiloid scale. Automatic calculation of the Centiloid scale has recently been available in some software packages (courtesy of Assistant Professor Chakmeedaj Sethanandha, Siriraj Hospital, Mahidol University).

The example formulas for calculating the Centiloid value generated for each radiopharmaceutical are as follows. However, the formula may vary depending on the data and the processing pipeline (e.g., SPM vs. PMOD vs. FSL Centiloid method) [78]. An example of cortical VOIs of beta-amyloid PET images coregistered with MRI is shown in Figure 6.

(1) CL_PiB_ = 93.7 × SUVR − 94.6
(2) CL_florbetapir_ = 175.43 × SUVR − 182.26
(3) CL_flutemetamol_ = 119.53 × SUVR − 118.57
(4) CL_florbetaben_ = 153.4 × SUVR − 154.9
(5) CL_NAV4694_ = 85.32 × SUVR − 87.97

Recent data with ^11^C-PiB and ^18^F-florbetaben using brain autopsy results as a gold standard found that a CL value < 10 accurately suggested no neuritic plaque, a CL value > 20 suggested at least moderate plaque density, and a CL value of 50 or more best confirmed both neuropathological and clinicopathological diagnosis of AD [79].

c. Three-dimensional stereotactic surface projection (3D-SSP) [80,81]

**Figure 7:**
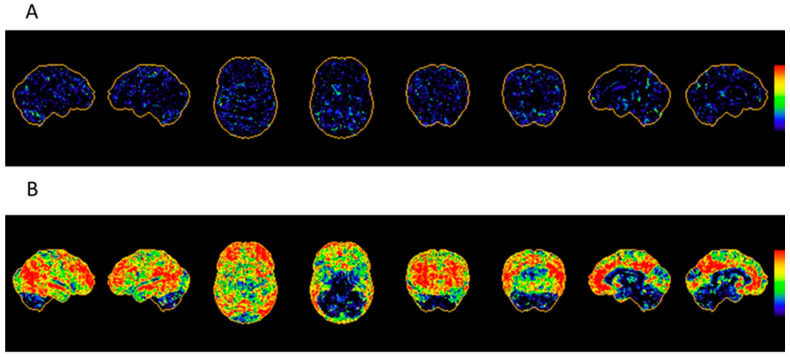
Examples of 3D-SSP Z-score map images from ^18^F-florbetapir PET scans in (**A**) normal elderly with negative amyloid deposition and (**B**) patients with Alzheimer’s disease and positive amyloid deposition. The 3D-SSP Z-score: Black represents negative amyloid deposition, progressively increasing deposition from blue to red on the color bar.

The 3D-SSP technique is used for assessing the difference in radiotracer activity in the brain in comparison with a normal database. The differences are displayed as Z-score and Z-score map images (Figure 7). This technique has been widely used with many radiopharmaceuticals, although the data in amyloid PET are still relatively limited [82,83].

#### 6.2.3. Reporting Format

Specific identification of each patient, the referring clinician, date and time of the study, and the reporting physician’s signature must be provided in the report as part of quality assurance. The responsibility of the reporting physician is to match this part to the body of the report correctly. The recommended structural body of the report includes four significant portions, as follows:

- History: The relevant history should be described along with an indication of the study. The specific clinical symptoms of MCI or dementia and the reasons for the test (e.g., atypical age of onset, uncertain clinical diagnoses, known comorbidities, or potential candidate for clinical trial) should be briefly documented.
- Techniques:
  1. Radiopharmaceutical type and dosage.
  2. Injection to imaging interval of radiopharmaceutical.
  3. Detailed imaging and processing techniques should be mentioned, including imaging quality and limitations. If certain specific software or anatomical co-registration is utilized, it should be additionally detailed.
  4. Type of additional software used for quantification analysis, e.g., 3D-SSP, SPM, PMOD, FreeSurfer, Hermes, MIMneuro, NeuroQ, or other commercially available or vendor-specific software
- Findings:
  - Visual analysis: The pattern of radiotracer distribution in the bilateral white matter and cerebellum should be discussed. The gray–white matter contrast should be mentioned, and the affected lobe with a loss of gray–white matter contrast should be noted. Abnormal radiotracer uptake in the cerebral cortices or cerebellar cortices, either the same degree and more intense than the white matter uptake, if any, should be described. If present, the degree and location of cerebral atrophy should also be mentioned.
  - Quantification analysis (optional): Describe the method used to obtain quantification data (SUVR or Centiloid scale) and the results.
- Interpretations/Impressions/Conclusions: Negative/positive brain PET scan for beta-amyloid deposition (absence/presence of significant beta-amyloid deposition in the brain).

Note: Indeterminate or inconclusive results should be reported with possible reasons, such as technical or physiological factors. The report should not state amyloid positivity as the diagnostic of Alzheimer’s disease [39,66].

**Evidence-based:**

Previous data revealed comparable sensitivity and specificity among different radiopharmaceuticals in differentiating patients with Alzheimer’s disease from normal controls [66,80,81]. The overall sensitivity and specificity of ^18^F-labeled amyloid tracers by visual analysis (pooled sensitivity of 90% and specificity of 82–95%) and quantification methods (pooled sensitivity of 90% and specificity of 83–94%) are also comparable. The pooled sensitivity of PiB PET is slightly higher (96%) but with lower specificity (58%) [24].

The recently reviewed data from several studies on the clinical utility of amyloid PET imaging in dementia disorders found that this technique is potentially proper on change of diagnosis in 29%, gain of diagnostic confidence in 63%, change of medication in 38%, and overall change of management in 64% of cases. The diagnostic confidence of referring physicians also increased by approximately 20% with amyloid PET results [84]. The management effect of amyloid PET in terms of change of diagnosis was also higher when the scan was performed under AUC (62.4%) than when a non-AUC scan was performed (45.2%) [85]. The impact of amyloid imaging on patient outcomes is still under investigation.

### 6.3. Tau PET

Tau is a protein that has the role of stabilization in the microtubules in neurons, predominantly within axons. If abnormality occurs, tau protein is hyperphosphorylated, which causes it to lose the function of binding to microtubules and then aggregate into neurofibrillary tangles (NFTs), leading to loss of cell function and cell death [86]. Hyperphosphorylated tau has six isoforms, which can be divided into two functional groups based on the number of repeated microtubule-binding domains [3R and 4R] [87].

Tauopathies are neurodegenerative disorders characterized by abnormal tau protein deposition in the brain, resulting in clinical syndromes [88]. Primary tauopathies constitute a significant class of frontotemporal lobar degeneration (FTLD) neuropathy and can have several presentations, such as frontotemporal dementia (FTD) [i.e., behavioral variant FTD, primary progressive aphasia], progressive supranuclear palsy (PSP), and corticobasal degeneration (CBD). In Alzheimer’s disease (AD), the primary pathology includes neurofibrillary tau neuropathy in addition to amyloid-beta (Aβ) and is considered a secondary or non-primary tauopathy (Table 11) [89,90,91].

In AD, neurofibrillary tangles (NFTs) are a key characteristic. The quantity of NFTs is linked to the severity of AD, indicating a stronger association with neuronal dysfunction compared to amyloid imaging. According to the Braak AD staging, NFTs initially appear in the perirhinal and entorhinal cortex (stage I), then in the CA1 region of the hippocampus (stage II). They subsequently accumulate in the limbic structures, including the hippocampus (stage III) and the amygdala, thalamus, and claustrum (stage IV). In the advanced stages, NFTs spread throughout the neocortex, with earlier and more severe impacts on specific areas (stage V) before affecting the primary sensory, motor, and visual regions (stage VI). Therefore, a tau pathology biomarker is a promising tool for diagnosing AD. In contrast to Alzheimer’s disease, there is still limited data for tau accumulation in the non-AD tauopathy group. However, some studies are showing that CBS patients have higher uptake in the putamen, globus pallidus, and subthalamic nucleus [92], and PSP patients also show significantly higher uptake in the subcortical brain region, especially in the globus pallidus but not in the dorsal midbrain [93].

For tau imaging, there are several types of tau radiopharmaceuticals, e.g., quinolone derivative (^18^F-THK 523, ^18^F-THK 5117, ^18^F-THK 5351), benzothiazole derivative (^11^C-PBB3), and benzimidazole derivative (^18^F-AV1451) [94]. However, the first generation of tau radiopharmaceuticals had high off-target binding to monoamine oxidase enzyme. Thus, the second generation was developed. Among the second generation of tau radiopharmaceuticals, 18F-PI2620 has been used in Thailand. 18F-PI2620 has been proven to have a high binding affinity to phosphorylated tau (both 3R and 4R isoforms) with absent off-target binding in AD and non-AD tauopathies [46,95].

#### 6.3.1. Imaging Data Display

PET images are automatically co-registered with each individual, using an automatic voxel of interest (VOI) based on the maximum probability following the automated anatomical labeling (AAL)-merged atlas. PET images are then registered to the T1-MRI. The standardized uptake value ratio (SUVR) of ^18^F-PI-2620 is analyzed for various brain regions, using the cerebellar gray matter (excluding the vermis and anterior lobe surrounding the vermis) as the reference region [48]. The processed images are displayed with the color scale in the axial view along the anterior commissure—posterior commissure line (AC–PC line), coronal view, and sagittal view.

#### 6.3.2. Image Interpretation for Tau PET

##### Visual Analysis

**Figure 8:**
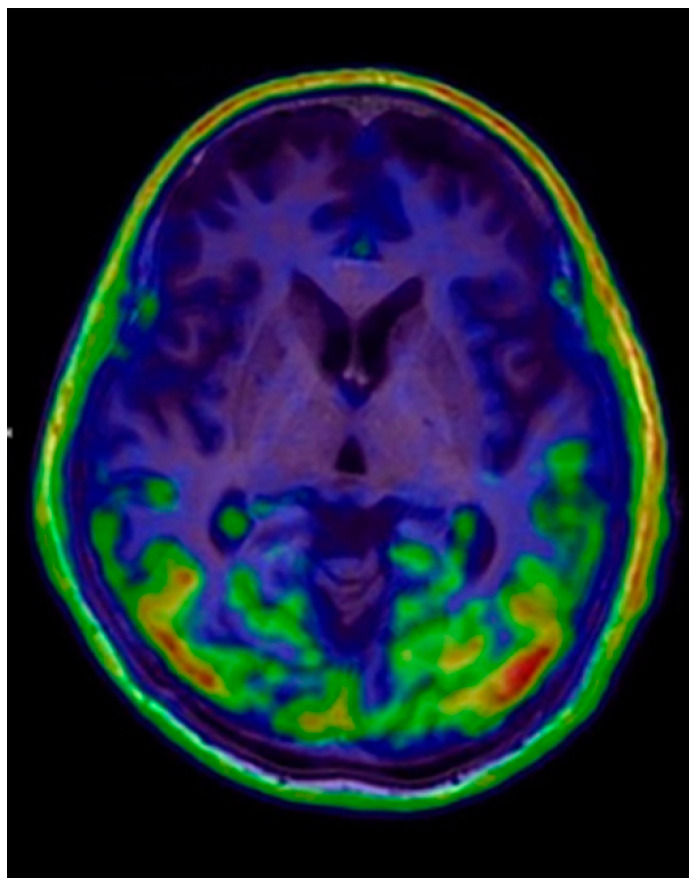
Positive tau PET: A fused ^18^F-PI2620 PET/T1-weight MRI image shows increased ^18^F-PI-2620 uptake at the inferior temporal, occipital, and parietal cortices and no off-target binding at the basal ganglia.

The normal population has no specific area of ^18^F-PI-2620 cerebral uptake visually. However, variable uptake of the skull may be generally seen. Suppose there is tau accumulation in cortical regions, mainly at the temporal and parietal lobe, precuneus, and posterior cingulate cortex. The study will be interpreted as positive, as shown in Figure 8 [46,48].

##### Quantitative Analysis

Quantitation using the SUVR is still limited for ^18^F-PI-2620 due to the small number of patients, unintended patient movement in later time frames, and small brain volume in some regions. Thus, the SUVR should be interpreted along with visual analysis [48].

#### 6.3.3. Reporting Format

- History: Indication, the patient’s clinical presentation, and correlative imaging.
- Techniques: Imaging is performed on an integrated 64-slice PET/CT scanner for the whole brain, with non contrast-enhanced CT for attenuation correction and localization in the transaxial, coronal, and sagittal planes. A 3D emission dynamic scan of the same area is acquired in a one-bed positionSemiquantitative calculations are performed using PMOD software with the automatic anatomical labeling (AAL)-merged atlas to generate automatic voxels of interest for different brain regions.
- Findings: Visual analysis: Describe abnormal tau deposition in the brain region.
- Interpretations/Impressions/Conclusions: Negative/positive studies should be mentioned when reporting the region of abnormal tau deposition.

Note: Imaging data should be interpreted along with available data from clinical context and correlative imaging.

## 7. Pitfalls

Many biological and technical factors affect image interpretation, as shown in Table 12 [38,40,47,96].

## 8. Radiation Exposure Risk Concern and Management

In nuclear medicine imaging for dementia, managing radiation doses is essential, especially in older patients. Although SPECT generates slightly more radiation than PET, risks are minimal for a single scan but can accumulate with multiple imaging sessions. PET, on the other hand, generally provides less radiation exposure than SPECT with more detailed images. Thus, it is valuable in repeated imaging. To mitigate the risk, clinicians should balance diagnostic needs with exposure risks. On the nuclear medicine side, it is recommended to use minimum effective tracer doses and consider alternatives like PET/MRI to reduce radiation further. Nuclear imaging radiation doses are generally low, but optimizing protocols helps to minimize cumulative risk while providing essential diagnostic insight.

## 9. Conclusions and Future Direction

The scope of applicability for these guidelines is aimed at hospitals with nuclear medicine departments. Since SPECT is generally more accessible and cost-effective compared to PET, SPECT can help to differentiate dementia subtypes (e.g., Alzheimer’s disease vs. vascular dementia) using perfusion tracers. In complex cases in which the diagnosis is still questionable, referring to higher-level centers with PET to perform specific tracers for Alzheimer’s pathology, such as amyloid and tau imaging, is recommended for more accurate dementia subtype differentiation. Tailoring the use of SPECT and PET in dementia diagnosis based on the hospital level could ensure both cost-effectiveness and optimal patient care across various healthcare settings.

In the near future, PET/MRI could become more prominent for their ability to simultaneously provide metabolic and structural insights. New tracers targeting amyloid, tau, and other proteins could allow for even earlier and more precise diagnosis. Machine learning for SPECT and PET image analysis holds promise for improved accuracy in dementia subtype differentiation and disease progression tracking.

## Figures and Tables

**Table 1:** Common types of dementia related to neurodegenerative diseases.

Alzheimer’s disease (AD)	
Alzheimer’s disease (AD) variants	Posterior cortical atrophy (PCA)
	Logopenic aphasia (PCA)
	Frontal variant AD (fvAD)
	Corticobasal syndrome (CBS) variant AD
	Familial AD
Non AD dementia	Lewy body dementia (LBD) - Dementia with Lewy bodies (DLB)
	- Parkinson’s disease dementia (PDD)
	Frontotemporal lobar degeneration (FTLD)
	- Behavioral variant FTLD (bvFTLD)
	- Primary progressive non-fluent aphasia (PNFA) - Semantic dementia (SD) Vascular dementia (VaD)

**Table 2:** Indications for PET and SPECT in dementia syndrome.

Investigations	Implication for Clinical Practice	Level of Evidence
To predict AD dementia conversion		
FDG PET	++	I [12,13]
Brain perfusion SPECT	+	I [13]
Amyloid PET	++	I [14,15,16]
Tau PET (use with amyloid PET)	+	I [15]
To support early-onset Alzheimer’s disease (EOAD) diagnosis		
FDG PET	++	I [17,18,19]
Brain perfusion SPECT	+	II [20,21]
Amyloid PET	++	I [17,22,23,24,25]
Tau PET	+	I [25] II [26,27,28,29,30]
To differential diagnosis of AD variants		
FDG PET	++	I [17,19]
Brain perfusion SPECT	+	II [20,21]
Amyloid PET	++	I [17,22,23,24]
Tau PET	+/−	II [27,28,29,30]
To differential diagnosis of AD vs. non-AD dementia		
FDG PET	++	I [17,22,23,29]
Brain perfusion SPECT	+	II [31,32]
Amyloid PET	++	I [18,19,28,30]
Tau PET	+	III [20,21,24,27]
To differential diagnosis of LBD vs. non-LBD		
FDG PET	++	I [33]
Brain perfusion SPECT	+	I [33] II [34]
DAT SPECT	++	II [12,35]
MIBG scan	++	II [12]
Tau PET (differentiate DLB and PCA)	++	II [12]

Note: ++ clinically useful investigation; + possibly useful investigation; +/− investigational;. Levels of evidence: Level I: High-quality prospective cohort study with adequate power or systematic review of these studies; Level II: Lesser quality prospective cohort, retrospective cohort study, untreated controls from an RCT, or systematic review of these studies; Level III: Case–control study or systematic review of these studies; Level IV: Case series; Level V: Expert opinion; case report or clinical example; or evidence based on physiology, bench research, or “first principles”.

**Table 3:** Patient preparation for brain perfusion SPECT and brain PET.

Brain perfusion SPECT	Review clinical history, physical examination, and anatomical images. Advise the patient to avoid stimulants affecting cerebral blood flow: Caffeine, cola, alcohol, and energy drinks; Smoking.
FDG PET	Review clinical history, physical examination, and anatomical images. Fast for at least 4 h. Advise the patient to stop taking brain stimulants and drugs, as follows: Caffeine, cola, alcohol, and energy drinks; Smoking and excessive exercise; Stop takin these drugs for 4–6 half-lives: cocaine, anesthetics (propofol, isoflurane, barbiturates), benzodiazepine, corticosteroids, anti-psychotics (haloperidol, chlorpromazine), and cholinesterase inhibitors (donepezil, rivastigmine). On the acquisition day, blood glucose must be checked, and the optimal blood glucose level should not exceed 160 mg/dL. If blood glucose exceeds 200 mg/dL, rescheduling should be considered. Good hydration is advised. During the FDG injection and uptake period, place the patient in a quiet, dimly lit room with eyes open and ears unplugged. Sedation can be used if needed with short-acting benzodiazepines, such as midazolam, just before image acquisition. Continuously monitor vital signs and pulse oximetry during acquisition.
Amyloid and Tau PET imaging	Review clinical history, physical examination, and anatomical images No drug is advised to be withdrawn. Fasting is advised for at least 4 h to prepare for sedation if needed.

**Table 4:** Radiopharmaceutical dosage.

Radiopharmaceuticals	Dose
^99m^Tc HMPAO	555–1110 MBq (15–30 mCi) Typical: 740 MBq (20 mCi)
^99m^Tc ECD	555–1110 MBq (15–30 mCi) Typical: 1110 MBq (30 mCi)
^18^F-FDG	185–740 MBq (5–20 mCi)
Amyloid imaging	
- ^18^F-Florbetapir	370 MBq (10 mCi)
- ^18^F-Flutemetamol	185 MBq (5 mCi)
- ^18^F-Florbetaben	300 MBq (8 mCi)
- ^11^C-PiB	300–370 MBq (8–10 mCi)
Tau tracer	
- ^18^F-PI2620	185–300 MBq (5–8 mCi)
- ^18^F-AV1451	370 MBq (10 mCi)
Variability in injected dose recommendation is based on differences in absorbed dose	

**Table 5:** Radiation dosimetry in SPECT studies.

Agent	Dose in MBq Unit (mCi)	Organ Receiving the Highest Dose mGy/MBq	Effective Dose mSv/MBq
^99m^Tc HMPAO	555–1110 (15–30)	0.034 (kidney)	0.0093 mSv
^99m^Tc ECD	555–1110 (15–30)	0.05 (bladder wall)	0.0077

**Table 6:** Radiation dosimetry in PET/CT studies in adults.

Agent	Dose in MBq Unit (mCi)	Organ Receiving the Highest Dose mGy/MBq	Effective Dose mSv/MBq
^18^F-FDG	185–740 (5–20)	0.13 (bladder wall)	0.019
^18^F-Florbetapir	370 MBq (10 mCi)	0.143 (gallbladder wall)	0.019
^18^F-Flutemetamol	185 MBq (5 mCi)	0.287 (gallbladder wall)	0.034
^18^F-Florbetaben	300 MBq (8 mCi)	0.137 (gallbladder wall)	0.019
^11^C-PiB	300 MBq (8 mCi)	44.80 ± 29.30 (gallbladder wall)	5.3
^18^F-PI2620	185–300 MBq	N/A	N/A
^18^F-AV1451	370 MBq	N/A	0.024

N/A: Data not available.

**Table 7:** SPECT and SPECT/CT acquisition and reconstruction parameters.

Agent	Waiting Time after Injection	Acquisition Time (min)
^99m^Tc ECD	15 min–6 h (optimum at 45 min)	30
^99m^Tc HMPAO	30 min–6 h (optimum at 90 min)	30
**Instrument**	**SPECT**	
**Collimator**	Fan beam or parallel hole (LEHR/LEUHR) *	
**Energy setting**	140 keV, 15–20% energy window	
**Zoom**	To gain at least a pixel size equal to 1/3 to 1/2 of the expected resolution	
**Nuclide**	^99m^Tc	
**Matrix Size**	≥128 × 128	
**Scan mode**	Step and shoot or continuous	
**Rotation per view**	≤3° (total of 360° rotation)	
**Time per view**	About 15–30 s/projection (total count 5 × 10^6^ counts)	
**Scatter correction**	Optional	
**Reconstruction**	3D-OSEM **/FBP	
**Slice thickness**	Possible 3–5 mm (for maximal pixel resolution)	
**(if SPECT/CT)**	**Scout/Surview/Topogram CT** (optional in some vendors)	
**CT voltage**	120–140 kV	
**CT current**	≤30 mA	
**(if SPECT/CT)**	**CT** (for attenuation correction)	
**CT voltage**	120–140 kV	
**CT current**	≤80 mA	
**Slice thickness**	3–5 mm	
**Acquisition steps for an individual patient** - Lie supine with arms down on the machine table. - Immobilize the head to reduce movement. - Center the head in the field of view. - Hold the head straight without tilting in the canthomeatal line perpendicular to the detector. - Use the smallest radius of rotation as much as possible or use an automated contour setting from the patient to the detector to ensure maximal image resolution.		
**SPECT processing** - Review of projection data in cine mode and sonogram for an initial determination of image quality, patient motion, and artifacts. - Reconstruct by ordered subset expectation maximization (OSEM) or filtered back projection (FBP) algorithm. - Select the type of low pass filter, i.e., Butterworth, Hamming or Hanning. - Optimize reconstructing parameters, i.e., cut-off, order, iterations, and subset, depending on the injected activity, resolution, and camera sensitivity. - Use either calculated (e.g., Chang’s method) or measured (e.g., Gadolinium source or CT scan) attenuation for attenuation correction. - Apply scatter correction (optional) to improve image signal-to-noise ratio. The most popular one is triple-energy-window correction.		

* Low-energy high resolution (LEHR) or low-energy ultra high resolution (LEUHR); ** Ordered subsets expectation maximization (OSEM) or filtered back projection (FBP); SPECT = Single-photon emission computed tomography; CT = Computed tomography; AC = Attenuation correction; keV = Kiloelectronvolt; sec = Second; mm = Millimeter; kV = Kilovolt; mA = Milliampere.

**Table 8:** PET/CT acquisition and reconstruction parameters.

Scout or Surview CT	
**CT voltage**	120 kV
**CT current**	≤30 mA
**CT**	
**Scan type**	Helical
**Rotation time**	0.75–1 s
**Matrix**	512
**Slice thickness (mm)**	3–5 mm
**Slice increments**	continuous
**Pitch**	≤1
**CT voltage**	120–140 kV
**CT current.time**	≤50 mAs (Low dose CT), ≤250 mAs (diagnostic CT)
**PET**	
**Energy setting**	511 keV, 15–20% energy window
**Glucose metabolic radiopharmaceutical and dose**	^18^F-Fluorodeoxyglucose (FDG) 185–740 MBq (5–20 mCi)
**Amyloid radiopharmaceutical and dose**	^18^F-Florbetapir 370 MBq (10 mCi) ^18^F-Flutemetamol 185 MBq (5 mCi) ^18^F-Florbetaben 300 MBq (8 mCi) ^11^C-PiB 300 MBq (8 mCi)
**Tau radiopharmaceutical and dose**	^18^F-PI-2620 185–300 MBq (5–8 mCi) ^18^F-AV1451 370 MBq (10 mCi)
**Uptake time**	^18^F-FDG 30–60 min ^18^F-Florbetapir 30–50 min ^18^F-Flutemetamol 60–120 min ^18^F-Florbetaben 45–130 min ^11^C-PiB 40–70 min ^18^F-PI2620 30–75 min ^18^F-AV1451 80 min
**Mode**	3D
**Scan direction**	Toward head
**Scan Duration (min/bed)**	^18^F-FDG 5–30 min
	^18^F-Florbetapir 10 min
	^18^F-Flutemetamol 10–20 min
	^18^F-Florbetaben 15–20 min
	^11^C-PiB 40–70 min
	^18^F-PI2620 45 min (5 min per frame)
	^18^F-AV1451 20 min
**Attenuation correction**	Yes (use CT)
**Scatter correction**	Yes
**Reconstruction**	Iterative (OSEM)
**Acquisition steps for an individual patient** - Lie supine with arms down on the machine table. - Set the patient’s head in a holder in the center of the field of view with the canthomeatal line in the vertical position. - Immobilize the head to reduce movement.	
**PET processing** - Preview images for patient motion and ensure PET and CT images are matched before performing CT attenuation correction. - Images are reconstructed in the transaxial plane of 128 × 128 (for semiquantitative analysis) or preferably 256 × 256 matrix size or more for better image resolution. - Typical transaxial pixel size is 2–3 mm and slice thickness is 2–4 mm. - A final image resolution may vary between 2.5 and 10 mm full-width at half maximum (FWHM), depending on the resolution of the PET system. - The reconstruction parameters can be varied. Please refer to the manufacturer’s recommendations for the best choices of iterations, subsets, and smoothness.	

PET = Positron emission tomography; CT = Computed tomography; AC = Attenuation correction; keV = Kiloelectronvolt; sec = Second; mm = Millimeter; kV = Kilovolt; mA = Milliampere; min = Minute.

**Table 9:** Specific hypoperfusion/hypometabolic patterns in each type of dementia.

Dementia Type	Hypoperfusion/Metabolic Cortical and Subcortical Regions	Preservation of Perfusion/Metabolism
Alzheimer’s disease (AD)	- Posterior cingulate gyri, precunei, parietal lobes, and superior/posterior temporal lobes (can be asymmetric) - Frontal lobes in advanced cases AD variants (more predominate in hypoperfusion/hypometabolic region) - Frontal variant (fvAD): orbitofrontal, medial frontal - Logopenic variant primary progressive aphasia (lvPPA): left temporoparietal predominate, extending toward more anterior temporal cortex - Posterior cortical atrophy (PCA): bilateral parieto-occipital lobes (usually right predominate) ± frontal eye field	- Primary sensory-motor cortices - Primary visual cortices - Basal ganglia, thalami
Dementia with Lewy bodies (DLB)	- Similar findings to AD plus primary visual cortex	- Posterior cingulate gyri (relatively preserved cingulate island sign)
Frontotemporal lobar degeneration (FTLD)	- Frontal and anterior temporal lobes - FTLD variants (more predominate in hyperperfusion/hypometabolic region): - Behavioral variant (bvFTD): medial prefrontal - Semantic (SD): anterior temporal lobes - Progressive non-fluent aphasia (PNFA): pre-Rolandic left lateral and medial frontal	- Posterior cingulate
Vascular dementia	- Various locations in cortical and subcortical gray matter and cerebellum	

Note: Correlation with anatomical imaging for interpretation is recommended.

**Table 10:** The recommended color scale and criteria for a positive scan use visual and semiquantitative analysis with the standardized uptake value ratio (SUVR) for each radiopharmaceutical to assess beta-amyloid deposition.

Radiopharmaceuticals	Color Scale	Criteria For Positive Scan	Cortical Region	SUVR Cut-Off for Positive Scan	Reference Region
^11^C-PiB	Color (Rainbow)	Binding in GM > WM	N/A	1.4–1.6	Cerebellar cortex
^18^F-florbetapir	Black/white (Reverse gray)	Loss of GM/WM contrast > 1 region	Temporal > occipital > prefrontal > parietal	1.1–1.34	Whole cerebellum
^18^F-flutemetamol	Color (Rainbow)	Increased GM uptake or loss of GM/WM contrast > 4 cortical and 1 subcortical regions	Frontal > posterior cingulate/precuneus > insula > temporal > striatum	0.58–0.62	Pons
^18^F-florbetaben	Black/white (Gray)	Increased GM uptake extending to the cortical margin > 4 cortical regions	Lateral temporal > frontal > posterior cingulate > parietal	1.43	Cerebellar cortex
^18^F-NAV4694	Color (Rainbow)	N/A *	N/A *	1.4–1.5	Cerebellar cortex

* N/A = not available (but similar criteria as in ^11^C-PiB are assumed); Apart from visual and quantification analyses, as previously mentioned, there are several ongoing studies on using artificial intelligence (AI), which is beyond the scope of the current guidelines.

**Table 11:** Histopathological appearance of tau isoforms and conformation in different tauopathies.

Tauopathy	Histopathology		Tau Isoform
	Electron Microscope	Light Microscope	
Primary tauopathies			
Progressive supranuclear palsy	SF (and TF)	Tufted astrocytes; globose tangles	4R
Corticobasal degeneration	SF (and TF)	Astrocytic plaques	4R
Argyrophilic grain disease	SF	Oligodendroglial coiled bodies; limbic argyrophilic grains	4R
Pick’s disease	TF (and SF)	Pick’s bodies	3R
Myotonic dystrophy	N/A	Neurofibrillary tangles	Short 0N3R
Secondary tauopathies			
Alzheimer’s disease	PHF (and SF)	Neurofibrillary tangles	3R and 4R
Down syndrome	PHF (and SF)	Neurofibrillary tangles	3R and 4R
Chronic traumatic encephalopathy	PHF (and SF)	Neurofibrillary tangles	3R and 4R
Niemann-Pick disease type C	PHF (and SF)	Neurofibrillary tangles	3R and 4R

3R: three repeated; 4R: four repeated; PHF: paired helical filaments; SF: straight filaments; TF: twisted filaments; N/A: data not available. Short 0N3R: the shortest isoform of fetal tau (where N denotes the number of N-terminal inserts and R is the number of microtubule-binding domains).

**Table 12:** Pitfalls and Errors.

**Perfusion SPECT**	
Biological factors	Technical factors
Unintended brain activity from external stimuli Interference effect on cerebral blood flow, e.g., sedative drug Anatomical variations	Misregistration artifact Inappropriate processing, e.g., the reconstruction method Non-continuous color and inappropriate plane of the image display Level of contrast and background subtraction Partial volume effect on the corrected image
**FDG PET**	
Biological factors	Technical factors
Unintended brain activity from external stimuli Interference effect on cerebral glucose metabolism, e.g., sedative drug or high serum glucose level Recent radio- or chemotherapy Brain glucose metabolic alteration due to the aging process	Misregistration artifact Inappropriate processing, e.g., reconstruction method Non-continuous color and inappropriate plane of the image display Level of contrast and background subtraction Partial volume effect on the corrected image
**Amyloid and Tau PET**	
Biological factors	Technical factors
Aging Cortical atrophy Encephalomalacia Extracerebral activity Non-specific uptake in the white matter and skull Off-target binding of tau tracer	Partial volume artifact Motion artifact Low dose of tracer injection Delayed scan time Under-smooth reconstruction
